# Overexpressed Spindlin1 relocalizes to the nucleoli during fixation

**DOI:** 10.1016/j.bbrep.2026.102625

**Published:** 2026-05-12

**Authors:** Falk Ike Fischer, Antigona Pervetica, Marie Moeser, Selina Morgenthal, Josephine Kugler

**Affiliations:** Department of Chemical and Product Safety, German Federal Institute for Risk Assessment (BfR), Berlin, Germany

**Keywords:** SPIN1, Fixation artifact, Formaldehyde, Nucleolus, Subcellular localization

## Abstract

The histone reader protein Spindlin1 has emerged as a pivotal player in tumorigenesis. Consequently, it became a major focus of research and numerous functions and interactions were proposed. In concert, varying subcellular distributions of Spindlin1 have been reported. However, the origins of these variations remain unclear. Many findings on Spindlin1 are rooted in a fixation-based methodology. Here, we show that formaldehyde fixation leads to nucleolar enrichment of mCherry tagged Spindlin1 and that nucleolar antibody detection of Spindlin1 depends on the targeted epitope. This suggests that part of the discrepancies in literature are based on methodological artifacts and demonstrates the need for complementary approaches.

## Highlights

• Formaldehyde based fixation leads to rapid apparent nucleolar enrichment of Spindlin1.
• Nucleolar detectability of Spindlin1 in immunocytochemistry depends on the targeted epitope.
• Artifact formation depends on the intrinsically disordered region (amino acids 1 to 50) of Spindlin1.

## 1 Introduction

Spindlin1 (SPIN1) is a protein of the spindlin/Y-linked spermiogenesis-specific transcript repeat (Spin/Ssty) family that is conserved in vertebrates [[1], [2], [3]]. It plays a vital role as a maternal transcript and during germ cell maturation [1,4]. Furthermore, ubiquitous and myoblast-specific knockouts in mice are fatal [5]. SPIN1 upregulation, on the other hand, has been extensively linked to cancer formation and progression (reviewed in Ref. [6]). SPIN1 is involved in meiotic [1,7] and mitotic spindle function, and consequently, in chromosome segregation and cell cycle control [8]. Additionally, it is a histone reader with transcriptional cofactor functions in rDNA transcription and essential signaling pathways including WNT, FASN, RET, and AKT signaling [[9], [10], [11], [12], [13]]. SPIN1 was also suggested to serve as a recruitment domain beyond the transcriptional context like modulating mRNA stability, p53 stability, or DNA damage repair [[14], [15], [16]].

SPIN1 is composed of 262 amino acids (aa), an N-terminal intrinsically disordered region (IDR, aa1-50) followed by three Tudor-like domains (TLD). The IDR was recently reported to enable phase separation [17]. It harbors a basic nuclear localization sequence (NLS) that was proposed to additionally drive nucleolar localization [9]. The TLDs mediate the histone methyl reader function [10,[18], [19], [20], [21], [22]]. TLD2 conveys high-affinity binding to H3K4me3 [18]. TLD1 and 3 form a composite pocket that enables weak binding of H3R8me2a [10,19,20] and H3K9me2/3 [21]. H4K20me3 and H4R23me are further potential targets of SPIN1 [22]. These supplementary interactions are proposed to enhance H3K4me3 binding by TLD2. Additionally, TLD3 serves as a coupling site for SPINDOC, further modulating histone binding and transcriptional coactivation [23,24]. SPIN1's phosphorylation sites were suggested to govern further interactions, and consequently its distributions [1,25]. In connection with its identified functions and interactions, different subcellular distributions of SPIN1 were reported (Supplementary Table 1). The predominantly cytosolic distribution observed in murine and porcine oocytes was recently identified to be based on SPIN1's recruitment to the subcortical maternal complex [26]. In somatic cells and derived cell lines, SPIN1 localizes mostly in the nucleus. The reported subnuclear distributions, however, vary greatly with respect to condensate formation and nucleolar enrichment. It is possible that SPIN1 may redistribute or form condensates in certain biological contexts. Proposed interaction partners of SPIN1, for instance are known to redistribute under certain conditions like oxidative stress [27,28]. Prolonged stress conditions have also been linked to the nucleolar retention of certain proteins and the formation of amyloid structures (A-bodies) within the nucleoli [29]. However, the biological relevance of the published discrepancies concerning SPIN1 remain to be addressed. Most authors used either GFP fusion proteins or immunocytochemistry against endogenous SPIN1. Tagging proteins of interest enables live-cell imaging, but inherently alters their expression levels, size, surface charge, interaction surfaces, tendency to oligomerize, etc. Immunofluorescence circumvents these alterations but includes invasive steps and depends on the adequacy of employed antibodies. Therefore, both approaches should generally be used in concert [30]. Few SPIN1 publications show live-cell images; often direct fluorescence was used in conjunction with DAPI counterstaining or co-immunofluorescence both requiring fixation. Recently, it was shown that fixation can affect the phase separation behavior [31] and nucleolar enrichment of certain chromatin-associated proteins [32]. Thus, we hypothesized that such experimental artifacts contribute to the variance in the reported distribution of SPIN1.

## 2 Material and methods

### 2.1 Cell culture

MCF7 (MCF7, HTB-22; ATCC), HEK293 (293, ACC-305; DSMZ), and 3T3 (NIH-3T3, donation from WWU Münster, Germany) cells were cultivated at 37 °C, 5% CO_2_ in DMEM (PAN-Biotech), supplemented with 10% (v/v) fetal calf serum, 2 mM l-glutamine, 100 U/ml penicillin and 100 μg/ml streptomycin (PAN Biotech), and passaged every 3-4 days after reaching 80% confluency. D3 cells (ES-D3, CRL-1934; ATCC) were maintained in their stem cell state using 2i medium. In detail, they were cultivated at 37 °C, 5% CO_2_ in gelatin-coated dishes with DMEM/High Glucose (Cytiva) supplemented with 15% (v/v) fetal calf serum (PANSera ES), 1% (v/v) GlutaMAX 100x (Gibco), 1% (v/v) MEM NEA 100x (PAN), 100 μM β-mercaptoethanol (Carl Roth), 3 μM CHIR99021 (Axon Medchem), 1 μM PD0325901 (Axon Medchem), 1000 U/ml LIF (Merck Milipore, ESG1106), 50 U/ml penicillin and 50 μg/ml streptomycin (PAN Biotech). They were passaged every 2-3 days. All cell lines used have been authenticated within the last three years of usage and were tested for mycoplasm every month.

### 2.2 Cloning

Coding sequences for human (NM_006717.3) and murine SPIN1 (NM_001283028.2) were isolated from MCF7 and D3 cDNA, respectively and incorporated into the pmCherry-N1 (Clontech PT3974-5 Cat. No. 632523) using *Nhe*I and *Xho*I. The shortened SPIN1 variants (SPIN1_1-50_, SPIN1_27-44_, SPIN1_50-262_) were generated from the murine variant. For the dark human SPIN1 construct *Nhe*I and *Not*I were used, removing the linker and mCh sequence. All constructs were validated via sequencing (Eurofins). For primers refer to Supplementary Table 2.

### 2.3 Transfection procedures

Cells were transfected using Lipofectamine2000 (Invitrogen). Briefly, 65,000 MCF7 cells were seeded per cm^2^ (μSlide-8-well, Ibidi) and incubated for 24 h. 150 ng Plasmid DNA were diluted in 25 μl Opti-MEM (Gibco) and combined with 0.75 μl Lipofectamin2000, prediluted in 25 μl Opti-MEM. After 15 min of incubation at room temperature and thorough mixing, the transfection mix was added to the cells for 24 h. Similarly, for immunofluorescence experiments 135,000 MCF7 cells were seeded on gelatin-coated glass cover slips in 24-well plates, and after 24 h the cells were transfected with 400 ng plasmid DNA in 100 μl Opti-MEM and 3 μl Lipofectamine in 100 μl Opti-MEM. The other cell lines were transfected accordingly, with adaptations to cell numbers to reach a sufficient but subconfluent density for imaging.

### 2.4 Fixation procedures

Cells were rinsed once with PBS and then incubated in the respective fixative for 20 min. The fixative was then discarded, followed by 3 rinses in PBS and imaging. For the different temperature conditions all solutions were either chilled on ice (−20 °C for ethanol and methanol) or heated to the indicated temperature. After application of the fixative, the slides were kept at the indicated temperature.

Formaldehyde fixation was performed using ROTI®Histofix (CarlRoth). Paraformaldehyde and glutaraldehyde fixatives were freshly prepared at neutral pH using PBS. The glyoxal fixative was prepared with 70.88% (v/v) water, 19.73% (v/v) ethanol, 7.83% (v/v) 40% glyoxal solution; 0.75% (v/v) acetic acid, and adjusted to pH = 5 with NaOH.

For the live-fixation experiments, 150 μl ROTI®Histofix (preheated to 37 °C) was carefully pipetted to the side of the respective wells containing cells and 150 μl medium during recording.

### 2.5 Treatments

For the ATP block, sodium azide and 2-deoxy-d-glucose (2-DG) diluted in cell culture medium were added to the cells reaching a final concentration of 50 mM NaN_3_ and 30 mM 2-DG. Cells were then incubated and monitored for 20 min, followed by 4% formaldehyde fixation. For oxidative stress, hydrogen peroxide was added to the cells to a final concentration of 2 mM for 30 min. In parallel, the same treatments were performed in PBS as a control. For heat shock, cells were incubated in cell culture medium at 43 °C and 5% CO_2_.

### 2.6 Immunofluorescence

Cells were washed once with PBS and fixed with ROTI®Histofix for 20 min at room temperature. After washing with PBS, the cells were permeabilized for 30 min in 0.5% (v/v) Triton X-100 in PBS, and blocked for 1 h in blocking solution (5% (v/v) goat serum and 2.5% (w/v) BSA in PBS) at room temperature. Primary antibody incubation (1:250) was performed overnight at 4 °C in blocking solution, followed by 3 rinses in PBS. Then secondary antibody incubation (1:250) was performed for 1 h at room temperature in blocking solution. Samples were rinsed 3x with PBS-T, incubated with Hoechst33258 (1:10,000) in PBS for 20 min followed by another rinse in PBS and mounting with GlycerMountingGel (Dako). The following antibodies were used: 12105-1-AP (Proteintech, α-SPIN1(total)), NBP2-55843 (Novus Biologicals, α-SPIN1(aa1-53)), STJ95750 (St John's Laboratory, α-SPIN1(aa80-160)), ab22758 (Abcam, α-Nucleolin) and ab150077 (Abcam, α-IgG-AlexaFluor®488).

### 2.7 Live-cell staining

For live-fixation, cells were incubated with 1:2000 dilution of Hoechst33342 *(Life Technologies)* in medium 15 min prior to recording.

For live-cell imaging, Amytracker480 (Ebba Biotech) was added to the medium at a final dilution of 1:250, 10-20 h prior to imaging. Immediately before imaging, cells were washed once in PBS. For fixed cells, Amytracker480 was applied in a 1:500 dilution in PBS for 20 min at room temperature.

### 2.8 Imaging

All imaging was done with a Zeiss LSM700, with a 63x objective and 405 nm, 488 nm, and 555 nm lasers. For live-cell imaging the “Incubation Insert P-Set 2000” (Pecon) was used to maintain 37 °C and 5% CO_2_ during imaging.

### 2.9 Analysis

Relative fluorescence intensity of nucleoli and nucleoplasm was measured using ImageJ 1.54f. The nucleoplasm without nucleoli and the nucleoli were manually outlined as regions of interest (ROI). Where hard to delineate, the corresponding phase contrast image or Hoechst-channel was used as guide. The pixel values were averaged for each ROI. Where several nucleoli per cell were measured, the respective ROI mean values were averaged. The starting points of fixation in the live-fixation experiments were set to the frame in which the Hoechst dye signal markedly increased. Further analysis was done in R. Image cropping, brightness adjustments, scale bars were done using ZEN 2.6.76.00000.

### 2.10 Statistical analysis

All experiments have been conducted at least three times unless otherwise stated and representative images have been taken for illustration. To assess statistical significance, the Student's *t*-Test was applied with Bonferroni adjustment in case of multiple comparisons, using the R package “rstatix”. Differences were considered significant when p < 0.05 (∗), p < 0.01 (∗∗), p < 0.001 (∗∗∗), and p < 0.0001 (∗∗∗∗).

## 3 Results

### 3.1 Formaldehyde fixation rapidly alters the apparent distribution of fluorescent protein tagged SPIN1 in dependence of its IDR

**Fig. 1:**
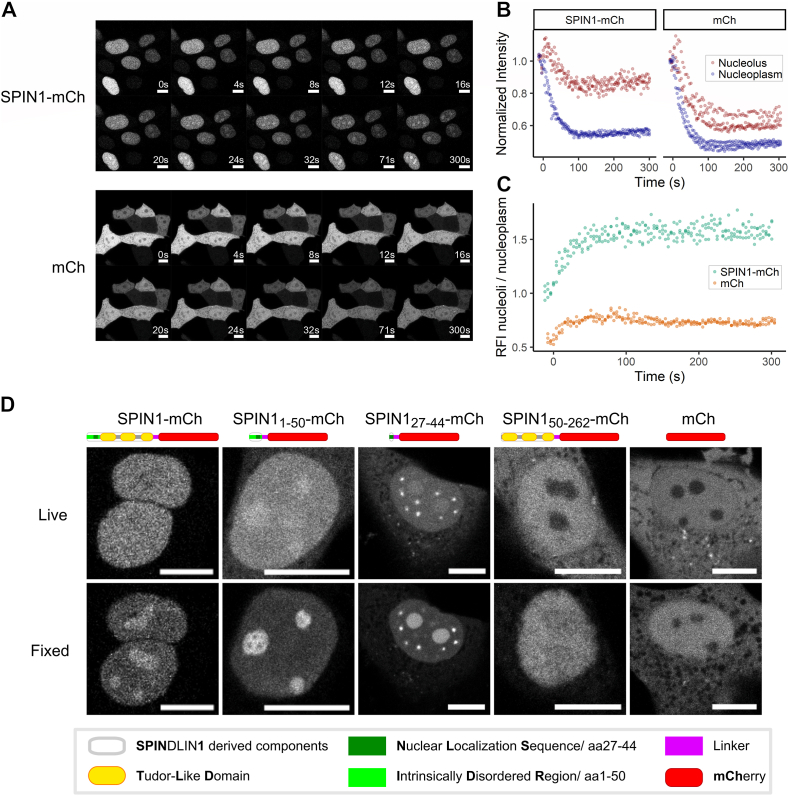
Formaldehyde fixation leads to rapid apparent nucleolar enrichment of mCh fusion proteins containing the SPIN1 N-Terminus. (A) Direct fluorescence imaging of MCF7 cells expressing SPIN1-mCh and mCh during the beginning of fixation with 2% formaldehyde. (B) Depicted are relative fluorescence intensity (RFI) of nucleoli and nucleoplasm after normalization to prefixation values over time. (C) Nucleolar-nucleoplasmic RFI ratios over time of SPIN1-mCh and mCh are shown. (D) Different SPIN1 mCh fusion proteins were analyzed before and after 4% formaldehyde fixation. Brightness of images was adjusted for better visibility and comparison. Scale bars represent 10 μm.

MCF7 cells transiently transfected with free mCherry (mCh) or SPIN1 fused to mCh were analyzed by confocal fluorescence imaging during formaldehyde application (Fig. 1A and Video 1 + 2). In live-cell conditions, SPIN1-mCh was homogenously distributed in the nucleus. Addition of formaldehyde rapidly induced nucleolar enrichment of SPIN1-mCh, while the mostly homogenous distribution of free mCh throughout the cell with partial exclusion from the nucleoli, remained largely unchanged. During fixation procedure, fluorescence intensity changed for both constructs. After an initial increase in intensity, presumably due to changes of the optical properties of the medium, intensity decreased by 20-50% within the first 100 s of fixation (Fig. 1B). The signal loss of SPIN1-mCh in the nucleoplasm occurred at similar rates as for mCh in the nucleoplasm and the nucleoli (Fig. 1B). In the nucleoli, however, SPIN1-mCh signal loss was markedly slower, resulting in an increase in relative fluorescence intensity (Fig. 1C).

We generated different mCh fusion proteins with the IDR (SPIN1_1-50_-mCh), the NLS (SPIN1_27-44_-mCh), and SPIN1 lacking the IDR (SPIN1_50-262_-mCh) to validate the IDR as driver behind nucleolar enrichment (Fig. 1D). In live-cell imaging, SPIN1_50-262_-mCh was distributed in the cytosol and nucleus, but excluded from the nucleoli. All other constructs were predominantly localized in the nucleus. The shortened variants SPIN1_1-50_-mCh and SPIN1_27-44_-mCh were markedly concentrated in the nucleoli, with SPIN1_27-44_-mCh also forming bright extranucleolar condensates.

Fixation markedly reduced fluorescence and changed the observed distributions of all constructs. The cytoplasmic signal decreased relative to the nuclear signal, which, in turn, decreased relative to the nucleolar signal, resulting in an apparent nuclear enrichment for SPIN1_50-262_-mCh and nucleolar enrichment for the constructs containing the NLS (Fig. 1D and Supplementary Fig. 1).

The distribution and fixation-associated shifts of SPIN1-mCh were also observed in transiently transfected HEK293, D3, and 3T3 cells and for the murine SPIN1-mCh (Supplementary Fig. 2). Similarly, SPIN1 tagged with GFP or YFP also redistributed upon formaldehyde application (data not shown). The presumed nucleolar localization was confirmed using an α-Nucleolin antibody (Supplementary Fig. 2).

### 3.2 Fixation protocol affects the extent of artifact formation

**Fig. 2:**
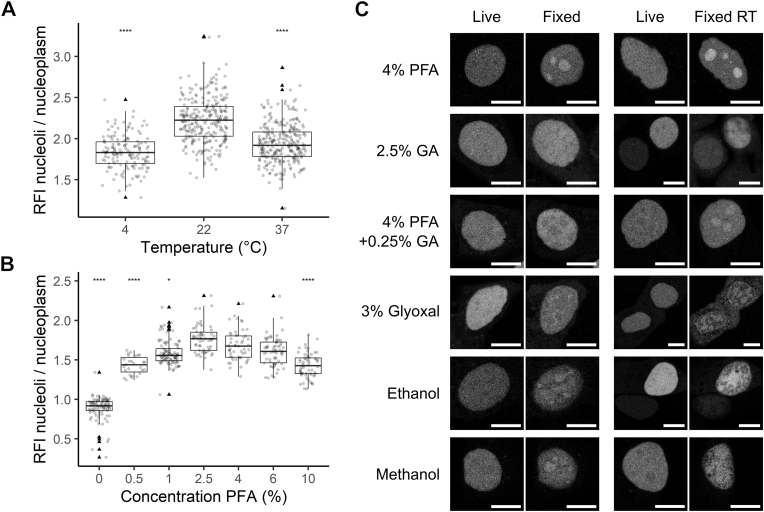
The fixation protocol affects artifact formation. (A) The ratio of mean intensity of SPIN1-mCh from nucleoli and nucleoplasm was calculated from individual MCF7 cells after fixation with 4% formaldehyde at 4 °C, 22 °C, and 37 °C and (B) after fixation with different concentration of paraformaldehyde at room temperature. Boxplots show the median and interquartile range. The whiskers indicate the 1.5x of the interquartile range. Triangles denote outliers. The dots represent the individual measurements. Statistically significant differences compared to 22 °C (A) or 4% (B) measurements are indicated with ∗ p < 0.05 and ∗∗∗∗ (p < 0,0001). (C) SPIN1-mCh transfected MCF7 cells were fixed with different fixatives at cold conditions (−20 °C for ethanol and methanol; on ice for the other fixatives) or room temperature. Scale bars represent 10 μm.

For fluorescence microscopy, numerous fixation protocols are utilized which might affect the observed outcomes [32]. Thus, we tested the effect of fixation temperature and fixative concentration and composition. SPIN1-mCh distribution was assessed by measuring the ratio between mean fluorescence intensity in the nucleoli and the nucleoplasm of single cells. In untreated cells, this ratio was close to 1. After 15 min fixation at room temperature with 4% formaldehyde, we detected the highest ratio of about 2. For 4 °C and 37 °C the ratio was markedly lower (Fig. 2A). Similarly, the observed nucleolar enrichment increased with the concentration of paraformaldehyde, peaked at 4% and then decreased again with higher concentrations (Fig. 2B). When testing different fixatives, artifact formation could be reduced but not completely abolished, as a small fraction (<5%) of cells still showed nucleolar enrichment under all conditions. For glyoxal, ethanol, and methanol, cell morphology was markedly altered after fixation, while glutaraldehyde produced autofluorescence. Generally, artifact formation and change of cellular morphology were less pronounced in cooled fixation (Fig. 2C).

### 3.3 Nucleolar detection of SPIN1 is antibody dependent

**Fig. 3:**
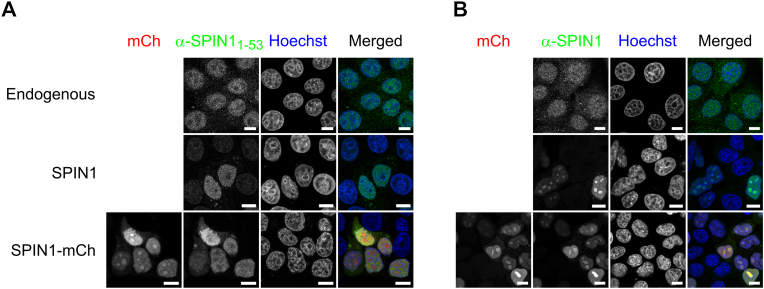
Nucleolar SPIN1 is not detectable with an antibody against the N-terminus. Depicted are immunostainings against SPIN1 with (A) an antibody raised against aa1-53 or (B) the entire protein of MCF7 cells transfected with no plasmid (endogenous), untagged SPIN1, or SPIN1-mCh. DNA was counterstained with Hoechst33258. Scale bars represent 10 μm.

To evaluate whether the results for SPIN1-mCh are reflective of endogenous SPIN1, we employed immunofluorescence with the fixation protocol that yielded the highest nucleolar enrichment. The first antibody tested (Fig. 3A) resulted in a nuclear signal excluding the nucleoli under endogenous conditions. To analyze the effect of overexpression, we transfected cells with untagged SPIN1. While much stronger in transfected cells, the antibody signal showed a similar nuclear distribution with nucleolar exclusion. Interestingly, the same was observed in cells expressing SPIN1-mCh. Direct fluorescence from SPIN1-mCh, however, showed the expected nucleolar enrichment (Fig. 3A). Otherwise, the immunofluorescence signal correlated well with the direct fluorescence of SPIN1-mCh. We therefore concluded, that while specific, the first antibody cannot detect SPIN1 in the nucleoli. Consequently, we tested two additional antibodies (Fig. 3B and Supplementary Fig. 3). Both stained endogenous SPIN1 throughout the nucleus, with homogenous or marginally stronger signal in the nucleoli compared to the nucleoplasm. In cells transfected with untagged SPIN1 or SPIN1-mCh, the signal was again much stronger but a clear nucleolar enrichment observable. Thus, the constructs mirror endogenous SPIN1 distribution, and overexpression with formaldehyde fixation causes nucleolar enrichment (Supplementary Fig. 1). The employed polyclonal antibodies were raised against different epitopes of SPIN1 (aa1-53, aa80-160, aa1-262), suggesting selective epitope masking of the IDR in the nucleoli.

### 3.4 Fixation colocalizes SPIN1-mCh with nucleolar amyloid structures but A-body formation associated stressors do not

**Fig. 4:**
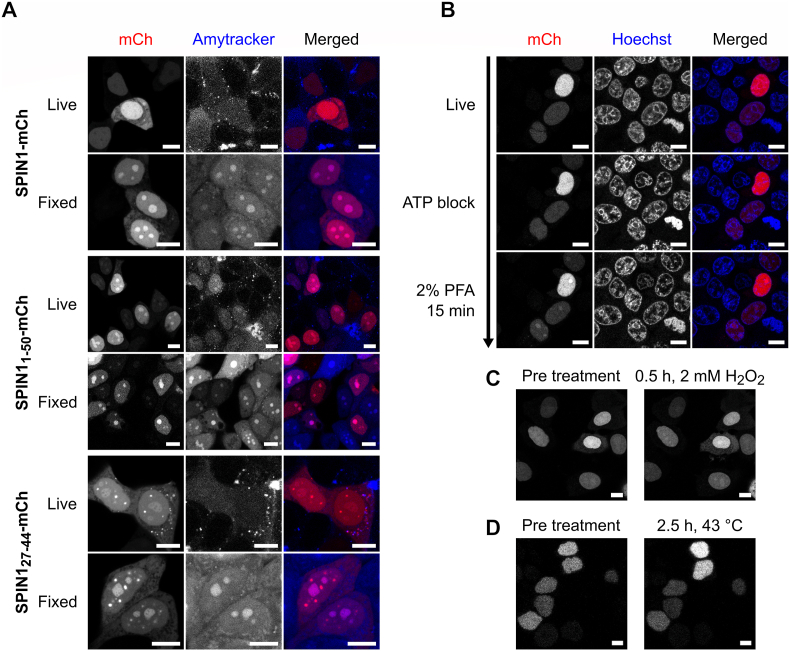
SPIN1-mCh relocalization coincides with enhanced nucleolar amyloid formation. (A) MCF7 cells expressing SPIN1-mCh, SPIN1_1-50_-mCh, and SPIN1_27-44_-mCh were live-stained with Amytracker480 and then fixed with 4% formaldehyde. (B) MCF7-cells expressing SPIN1-mCh were treated with 50 mM sodium azide and 30 mM 2-deoxy-d-glucose followed by formaldehyde fixation, (C) exposed to 2 mM hydrogen peroxide, or (D) heat shocked. Scale bars represent 10 μm.

To explore a potential involvement of A-bodies in artifact formation, we used Amytracker480, a dye specifically staining amyloid structures. Transfected MCF7 were incubated with the dye for 12 h for live-cell imaging. The Amytracker480 signal was generally weak and diffuse (Fig. 4A). Only in very few of the cells expressing the nucleolar SPIN1_1-50_-mCh and SPIN1_27-44_-mCh, the Amytracker480 signal was marginally enhanced in the nucleoli. In formaldehyde fixed cells, the Amytracker480 produced a much stronger signal, presumably due to better penetration or retention. Crucially, this signal was clearly concentrated in the nucleoli in all cells independent of transfection. Notably in the extranucleolar condensates formed by SPIN1_27-44_-mCh, the Amytracker480 signal did not, or only marginally, exceed the nucleoplasmic background. We concluded that formaldehyde fixation leads to amyloid like structures in the nucleoli, but a link between those structures and nucleolar enrichment of SPIN1 remained ambiguous.

To test whether SPIN1 nucleolar enrichment might be an active stress response, we performed an ATP block. This had no visible effect on the distribution of SPIN1-mCh in living cells and did not impair nucleolar enrichment in the subsequent formaldehyde fixation (Fig. 4B). Further, it did not affect the *in vivo* nucleolar enrichment of SPIN1_1-50_-mCh. However, it enhanced the Amytracker480 signal in the nucleoli over time (Supplementary Fig. 4), which is in line with the notion that the clearance of amyloids is partially ATP dependent [33].

We then tested oxidative stress and heat shock under live-conditions (Fig. 4C/D). Hydrogen peroxide treatment only induced a slight nucleolar enrichment in a small fraction of cells and a 2.5 h incubation at 43 °C did not alter SPIN1-mCh distribution. In comparison with other proteins, this weak or absent response to stressors commonly associated with nucleolar retention [29,34] does not support a principal role for SPIN1 in the nucleolar stress response. The rapid and extensive nucleolar enrichment during fixation, therefore, rather represents a physicochemical phenomenon than an active biological process.

## 4 Discussion

In this study, we show that the employed fixation strategy and antibody choice can alter the observed distribution of SPIN1 fusion proteins, which explains parts of the variance in the SPIN1 literature. Formaldehyde fixation can lead to rapid apparent nucleolar enrichment of SPIN1, depending on its IDR. Antibodies directed against the IDR, might fail to detect SPIN1 in the nucleoli.

Our mCh fusions replicated previous findings with similar GFP constructs [9] and support SPIN1's N-terminus as the driver for its nucleolar enrichment. By providing live-cell images, however, we reveal that this enrichment depends on formaldehyde fixation and overexpression. SPIN1_1-50_-mCh and SPIN1_27-44_-mCh showed nucleolar enrichment, and for the latter, also condensate formation *in vivo*. These constructs represent a concentration of the IDR and positively charged patches, furthering the notion that nucleolar localization and condensation are driven by overall physicochemical properties rather than specific sequences [35,36]. This also highlights the possibility that minor differences in the employed reporters (e.g., linker sequence or position of the tag) affected different live-cell distributions of GFP-tagged SPIN1 in literature (compare Supplementary Table 1). To rule out potential effects of the tag, it is common practice to turn to immunofluorescence. However, we show that the employed fixation protocol is likely to affect SPIN1's distribution, further complicating validation.

Our results suggest an association between formaldehyde fixation and A-body formation. Further we conclude that the redistribution of SPIN1-mCh during fixation is likely not a physiological response, considering its speed and extent contrasted by the weak response to the tested stressors. Particularly in overexpression, SPIN1 binding sites in the nucleoplasm may be saturated or lost through fixation [32]. Given its high mobility [37], unbound nucleoplasmic SPIN1 might initially escape fixation and diffuse [38]. In the distinct nucleolar environment, increased viscosity, molecular crowding [39], and potentially non-specific binding sites provided in amyloid structures may slow SPIN1 and enable preferential nucleolar fixation. The flexible IDR is likely to adopt new conformations. In context with condensation and aggregation, this might predispose it for incorporation in A-bodies, explaining the diminished nucleolar detection we observed with the first antibody.

Deciphering whether nucleolus specific enrichment and epitope masking are linked to A-body formation, will require validation of SPIN1's incorporation. Tracking the net flux of SPIN1 will aid in assessing whether other mechanisms, such as compartment specific washing out or alteration of fluorophore properties, contributed to the observed relative nucleolar enrichment.

In conclusion, this study adds to the mounting evidence that fixation does not necessarily provide an accurate “snapshot” of the *in vivo* localization of proteins. Alternative methods are needed to reconcile the published findings and derive SPIN1's physiological subnuclear distribution. Approaches avoiding fixation e.g. enzyme-tethering-based approaches such as ChromID [40] or DamID [41], could aid in reassessment of the published immunoprecipitation and ChIP-seq experiments. Another supplemental approach might be the fractionation of nucleoli and nucleoplasm, followed by mass spectrometric analyses [42] to bypass or further study artifacts by fixation, overexpression, and modification of SPIN1.

## CRediT authorship contribution statement

**Falk Ike Fischer:** Conceptualization, Formal analysis, Investigation, Writing – original draft. **Antigona Pervetica:** Investigation. **Marie Moeser:** Investigation. **Selina Morgenthal:** Investigation. **Josephine Kugler:** Conceptualization, Formal analysis, Supervision, Writing – review & editing.

## Declaration of competing interest

The authors declare the following financial interests/personal relationships which may be considered as potential competing interests: Falk Ike Fischer reports financial support was provided by Horizon Europe. If there are other authors, they declare that they have no known competing financial interests or personal relationships that could have appeared to influence the work reported in this paper.

## Data Availability

Data sets can be found under following link: doi: 10.17632/mck6z3xyj6.1
