# Supplementary material for: Overexpressed Spindlin1 relocalizes to the nucleoli during fixation
Source: Biochem Biophys Rep. 2026 May 12;46:102625. doi: 10.1016/j.bbrep.2026.102625 (PMC13195701; doi:10.1016/j.bbrep.2026.102625)
Supplement: Multimedia component 1 [file mmc1.docx]

**Supplements**

Overexpressed Spindlin1 relocalizes to the nucleoli during fixation

Falk Fischer, Gona Berisha, Marie Moeser, Selina Morgenthal and Josephine Kugler*

Department of Chemical and Product Safety, German Federal Institute for Risk Assessment (BfR),
Berlin, Germany

* Corresponding author.

E-mail address:  josephine.kugler@bfr.bund.de

| **Supplementary Table 1** Spindlin1 distributions in published literature | | | |
| --- | --- | --- | --- |
| Distribution | Live, direct fluorescence | Fixed, direct fluorescence | Immunofluorescence |
|  |  | [1]:**GFP-SPIN1;**  [2]:**GFP-SPIN1** | [3];  [4];  [5]:primary gibel carp oocytes;  [2]:**Myc-SPIN1** α-myc |
|  |  | [6]:**eGFP-SPIN1** | [6];  [7] |
|  |  | [3]:**GFP-SPIN1**;  [4]:**SPIN1-GFP**, SPIN1-F141A-GFP, SPIN1-W72R/Y98R-GFP, SPIN1_1-49_-GFP, SPIN1_28-49_-GFP, SPIN1_28-39_-GFP;  [8]:**SPIN-GFP**, formalin; | [1]:**Myc-SPIN1** α-myc; |
|  | [6]:**eGFP-SPIN1**;  [9]:GFP-SPIN1_Δ125–190_, GFP-SPIN1_Δ190–262_ | [6]:**eGFP-SPIN1**, **SPIN1-eGFP**;  [4]:SPIN1_28-44_-GFP | [7];  [9] |
|  | [10]:**GFP-SPIN1**, GFP-SPIN1-F141A;  [11]:**Halo-SPIN1**;  [9]:GFP-SPIN1_Δ51–125_ | [12]:**GFP-SPIN1**;  [4]:SPIN1_1-49_-5A-GFP, SPIN1_28-35_-GFP;  [13]:eGFP-SPIN1_26-262_ 3.7% (v/v) polyoxymethylene;  [14]:SPIN1_26-239_-EGFP, SPIN1_26-253_-EGFP, SPIN1_26-262_-EGFP acetone | [15];  [5]:gibel carb oocytes stages III and IV |
|  | [6]:**eGFP-SPIN1**;  [9]:**GFP-SPIN1** | [6]:**eGFP-SPIN1** |  |
|  | [16]:**eGFP-SPIN1**, 1-4 cell stadium porcine embryos;  [9]:GFP-SPIN1_Δ1–50_ | [17]:**eGFP-SPIN1**;  [14]:SPIN1_26-262_-eGFP;  [13]:eGFP-SPIN1_26-262_ 3.7% (v/v) polyoxymethylene;  [6]:SPIN1_Δ1–20_-eGFP, eGFP-SPIN1_Δ1–20_, eGFP-SPIN1_Δ1–30_, eGFP-SPIN1_Δ1–40_, eGFP-SPIN1_Δ1–50_,  [4]:SPIN1_50-262_-GFP, SPIN1-5A-GFP;  [18]:eGFP-SPIN1-S14A, eGFP-SPIN1-S84A | [19] |
|  | [6]:eGFP-SPIN1_Δ1–50_ | [18]:**eGFP-SPIN1**, eGFP-SPIN1-S99A,  eGFP-SPIN1-S14A/S99A;  [6]:eGFP-SPIN1_Δ1–10_ , SPIN1_Δ1–10_-eGFP, GFP-SPIN1-5A |  |
|  | [20]:**mCherry–SPIN1** | [18]:EGFP-SPIN1-S14A/S84A, EGFP-SPIN1-S84A/S99A, EGFP-SPIN1-S14A/S84A/S99A;  [13]:eGFP-SPIN1_26-262_ 3.7% (v/v) polyoxymethylene;  [14]:SPIN1_26-239_-eGFP, SPIN1_34-262_-eGFP, SPIN1_54-262_-eGFP acetone;  [6]:SPIN1_Δ1–30_-eGFP, SPIN1_Δ1–40_-eGFP, SPIN1_Δ1–50_-eGFP |  |
|  |  | [14]:SPIN1_26-239_-eGFP;  [6]:SPIN1_Δ1–30_- eGFP |  |
|  | [20]:**mCherry–SPIN1** murine oocytes GV |  | [20]:murine oocyte GV,2 cell, morula;  [21]:developing murine oocytes |
|  |  |  | [16] porcine MII stage oocytes to 4 cell stage embryos methanol;  [22]:porcine MI, MII stage oocytes methanol |
| The classification was done based on the predominant distribution of SPIN1 in the provided micrographs. Unless otherwise denoted: (1) the investigated cells where somatic cell lines or tissues, (2) immunofluorescence was performed against endogenous Spindlin1, and (3) the fixative was a 4% PFA solution or in some cases not specified. For publications showing DAPI staining or immunocytochemistry without specified methodology, we assumed fixation. We highlighted constructs with full length, unmutated SPIN1 in bold fond. 5A denotes the mutations of the NLS (K28A/K29A/R30A/K34A/K35A). The distributions of SPIN1 during mitosis [3, 13, 17, 23]. | | | |

1.	Du, Y., et al., *Structural mechanism of bivalent histone H3K4me3K9me3 recognition by the Spindlin1/C11orf84 complex in rRNA transcription activation.* Nat Commun, 2021. **12**(1): p. 949.

2.	Fang, Z., et al., *SPIN1 promotes tumorigenesis by blocking the uL18 (universal large ribosomal subunit protein 18)-MDM2-p53 pathway in human cancer.* Elife, 2018. **7**.

3.	Wang, W., et al., *Nucleolar protein Spindlin1 recognizes H3K4 methylation and stimulates the expression of rRNA genes.* EMBO Rep, 2011. **12**(11): p. 1160-6.

4.	Zhang, X., et al., *Nucleolar localization signal and histone methylation reader function is required for SPIN1 to promote rRNA gene expression.* Biochem Biophys Res Commun, 2018. **505**(1): p. 325-332.

5.	Sun, M., Z. Li, and J.F. Gui, *Dynamic distribution of spindlin in nucleoli, nucleoplasm and spindle from primary oocytes to mature eggs and its critical function for oocyte-to-embryo transition in gibel carp.* J Exp Zool A Ecol Genet Physiol, 2010. **313**(8): p. 461-73.

6.	Wang, Y., et al., *Phase separation of SPIN1 through its IDR facilitates histone methylation readout and tumorigenesis.* J Mol Cell Biol, 2024. **16**(6).

7.	Zhao, M., et al., *SPIN1 triggers abnormal lipid metabolism and enhances tumor growth in liver cancer.* Cancer Lett, 2020. **470**: p. 54-63.

8.	Lui, J.C., et al., *Loss-of-function variant in SPIN4 causes an X-linked overgrowth syndrome.* JCI Insight, 2023. **8**(9).

9.	Wang, Y., et al., *SPIN1 facilitates chemoresistance and HR repair by promoting Tip60 binding to H3K9me3.* EMBO Rep, 2024. **25**(9): p. 3970-3989.

10.	Yapp, C., et al., *Frapid: achieving full automation of FRAP for chemical probe validation.* Biomed Opt Express, 2016. **7**(2): p. 422-41.

11.	Liu, X., et al., *Driving integrative structural modeling with serial capture affinity purification.* Proc Natl Acad Sci U S A, 2020. **117**(50): p. 31861-31870.

12.	Bae, N., et al., *A transcriptional coregulator, SPIN.DOC, attenuates the coactivator activity of Spindlin1.* J Biol Chem, 2017. **292**(51): p. 20808-20817.

13.	Zhang, P., et al., *Overexpression of spindlin1 induces metaphase arrest and chromosomal instability.* J Cell Physiol, 2008. **217**(2): p. 400-8.

14.	Gao, Y., et al., *Spindlin1, a novel nuclear protein with a role in the transformation of NIH3T3 cells.* Biochem Biophys Res Commun, 2005. **335**(2): p. 343-50.

15.	Greschik, H., et al., *The histone code reader Spin1 controls skeletal muscle development.* Cell Death Dis, 2017. **8**(11): p. e3173.

16.	Choi, J.W., et al., *Spindlin 1 is essential for metaphase II stage maintenance and chromosomal stability in porcine oocytes.* Mol Hum Reprod, 2017. **23**(3): p. 166-176.

17.	Yuan, H., et al., *Overexpression of SPINDLIN1 induces cellular senescence, multinucleation and apoptosis.* Gene, 2008. **410**(1): p. 67-74.

18.	Cong, B., et al., *Ser84 is The Key Point of Spindlin1 Nuclear Localization and Function.* Progress in Biochemistry and Biophysics, 2009. **36**(2): p. 175-181.

19.	Stenstrom, L., et al., *Mapping the nucleolar proteome reveals a spatiotemporal organization related to intrinsic protein disorder.* Mol Syst Biol, 2020. **16**(8): p. e9469.

20.	Xu, C., et al., *The subcortical maternal complex safeguards mouse oocyte-to-embryo transition by preventing nuclear entry of SPIN1.* Nat Struct Mol Biol, 2025. **32**(8): p. 1358-1372.

21.	Chew, T.G., et al., *A tudor domain protein SPINDLIN1 interacts with the mRNA-binding protein SERBP1 and is involved in mouse oocyte meiotic resumption.* PLoS One, 2013. **8**(7): p. e69764.

22.	Choi, J.W., et al., *Spindlin1 alters the metaphase to anaphase transition in meiosis I through regulation of BUB3 expression in porcine oocytes.* J Cell Physiol, 2019. **234**(6): p. 8963-8974.

23.	Wang, J.X., et al., *SPINDLIN1 promotes cancer cell proliferation through activation of WNT/TCF-4 signaling.* Mol Cancer Res, 2012. **10**(3): p. 326-35.

**Supplementary Fig. 1.** Quantification of artefact formation. (A) Mean relative fluorescence intensity of nucleoli against surrounding nucleoplasm of different SPIN1-constructs before and after fixation for individual MCF7 cells corresponding to Fig. 1D and Supplementary Fig. 2B (n≥15). (B) Mean relative fluorescence of nucleoli against surrounding nucleoplasm for three different antibodies against SPIN1 under endogenous conditions and in MCF7 cells transfected with SPIN1 or SPIN1-mCh, respectively (n≥15). (C) Comparison of mean relative fluorescence of nucleoli against surrounding nucleoplasm between antibody staining (g) and direct fluorescence of SPIN1-mCh (r) in individual cells (n≥15). B and C are corresponding to Fig. 3 and Supplementary Fig 3. Boxplots show the median and interquartile range. The whiskers indicate the 1.5x of the interquartile range. The dots represent the individual measurements. Gray lines show paired measurements of individual cells in different states (A) or different channels (C), respectively.

**Supplementary Fig. 2.** Formaldehyde fixation leads to nucleolar enrichment of SPIN1-mCh and mSPIN1-mCh independent of cell type. (A) 3T3, D3, and HEK293 cells expressing SPIN1-mCh before and after 4% formaldehyde fixation. (B) MCF7 cells expressing murine mSPIN1-mCh before and after 4% formaldehyde fixation. (C) Anti-Nucleolin immunostainings and direct fluorescence of MCF7 cells expressing SPIN1-mCh or SPIN1_27‑44_‑mCh. DNA was counterstained with Hoechst33258. Scale bars represent 10 µm.

**Supplementary Fig. 3.** Nucleolar SPIN1 is detectable with an antibody against the central part. Depicted are anti-SPIN1 (aa80-160) immunostainings of MCF7 cells transfected with no plasmid (endogen), SPIN1, or fluorescent SPIN1-mCh. DNA was counterstained with Hoechst33258. Scale bars represent 10 µm.

**Supplementary Fig. 4.** A prolonged ATP block does not change the distribution of SPIN1_1-50_‑mCh but enhances A-bodies. MCF7 cells transfected with SPIN1_1-50_‑mCh were live-stained with Amytracker480 and imaged during a 50 mM sodium azide and 30 mM 2-deoxy-D-glucose ATP block. The micrographs show representative cells. The experiment was performed twice as technical replicate. Scale bars represent 10 µm.

| **Supplementary Table 2** Primers for introduction of restriction sites and isolation of SPIN1 derivatives | |
| --- | --- |
| Constructs | Primer Pair |
| SPIN1-mCh/mSPIN1-mCh | CTGAGCTAGCCACCATGAAGACCCCATTCG CGATCTCGAGGGATGTTTTCACCAAATCGTA |
| SPIN1 | CTGAGCTAGCCACCATGAAGACCCCATTCG  ATGCGGCCGCTAGGATGTTTTCACCAAATCGTA |
| SPIN1_1‑50_‑mCh | CTGAGCTAGCCACCATGAAGACCCCATTCG  CGATCTCGAGGGGTTGGGACACAGGCTTGC |
| SPIN1_27‑44_‑mCh | CTGAGCTAGCCACCATGAAGAAGAGGACATCTCAC  CGATCTCGAGCTTGCTTGGTCCCACA |
| SPIN1_50-262_‑mCh | GACTGCTAGCCACCATGCGGCGGAACATCGTAGGC  CGATCTCGAGGGATGTTTTCACCAAATCGTA |
